# Numerical Investigation on Static and Rotor-Dynamic Characteristics of Convergent-Tapered and Divergent-Tapered Hole-Pattern Gas Damper Seals

**DOI:** 10.3390/ma12142324

**Published:** 2019-07-21

**Authors:** Dan Sun, Sheng-Yuan Li, Huan Zhao, Cheng-Wei Fei

**Affiliations:** 1 Liaoning Key Lab of Advanced Test Technology for Aerospace Propulsion System, Shenyang Aerospace University, Shenyang 110136, China; 2 School of Power and Energy, Northwestern Polytechnical University, Xi’an 710072, China; 3 Department of Aeronautics and Astronautics, Fudan University, Shanghai 200433, China

**Keywords:** hole-pattern damper seals, taper seal clearance, static characteristics, rotor-dynamic characteristics, rotor stability

## Abstract

To study the influence of taper seal clearance on the static and rotor-dynamic characteristics of hole-pattern damper seals, this paper develops three-dimensional transient computational fluid dynamic methods, which comprise single-frequency and multi-frequency elliptical orbit whirl model, by the transient solution combined with a mesh deformation technique. Through the investigations, it is illustrated that: (1) In the present paper, the leakage rates of convergent-tapered hole-pattern damper seals are less than divergent-tapered hole-pattern damper seals for the same average seal clearance, and the maximum relative variation reaches 16%; (2) Compared with a constant clearance hole-pattern damper seal, the maximum relative variation of the rotor-dynamic coefficients is 1,865% for nine taper degrees in this paper; (3) Convergent-tapered hole-pattern damper seals have smaller reaction forces and effective damping coefficient, larger cross-over frequency, and direct stiffness coefficient, while divergent-tapered damper seals have the opposite effects; (4) Divergent-tapered hole-pattern damper seals alleviate the rotor whirl because of a larger effective damping coefficient when the rotor system has large natural frequency and small eccentricity. Convergent-tapered damper seals provide both sealing and journal bearing capabilities at the same time, and are more advantageous to the stability of the rotor system when rotor eccentricity is the main cause of rotor instability.

## 1. Introduction 

Annular gas seals in turbomachinery are widely used to minimize the leakage rate through clearance involved with rotating and stationary parts [1]. The advanced seal design can improve the efficiency of turbomachinery and the stability of the rotor system [2]. However, currently, the design of advanced turbomachinery requires higher speed, tighter clearance, and higher thrust to weight ratio. In this situation, rotor instability has become more critical due to the reaction force in annular gas seals [3]. Damper seals such as honeycomb and hole-pattern seals are attractive from a rotor-dynamic standpoint since they tend to have higher effective damping, and a better leakage rate control than see-through labyrinth seals [4].

Labyrinth seals are the most classic design of annular gas seals, comprising of a series of circular blades and annular grooves. Labyrinth seals have simplicity, reliability, and tolerance for large thermal and pressure variation [5,6]. However, numerous investigations have found that the labyrinth seals in turbomachinery are an important source of inducing rotor instability [7,8]. To overcome this issue, damper seals were proposed in the 1960s, and then it was validated to hold good performance in the control of turbomachinery vibration in the 1980s [9]. Soto and Childs [10] quantitatively revealed the positive effects of honeycomb damper seals on the rotor-dynamic stability of the rotor system. However, the honeycomb damper seal is normally manufactured by brazing, and has the disadvantages of complex manufacturing and rub causing rotor damage. The hole-pattern damper seal is made by milling holes on a smooth stator surface, and, thereby, may avoid the disadvantages of the honeycomb damper seal [11]. Yu et al. [12] tested the rotor-dynamic coefficients of three hole-pattern damper seals, and found that the performance of hole-pattern damper seals with a 69% hole-area ratio was slightly lower than the competitive honeycomb damper seal. 

Currently, damper seals have been studied extensively through experimental measurements and numerical modeling. Ha et al. [13,14] measured friction factors from a flat plate test system for various configurations of honeycomb damper seals including the depths of three holes. Childs et al. [15,16] experimentally measured the impact of whirl frequency of the rotor, pre-swirl, and hole depth on the leakage and rotor-dynamic characteristics of hole-pattern damper seals. Relative to experimental measurements, numerical modeling has the advantages of economy and convenience in the design of annular gas seals. There are two effective numerical modelings including bulk-flow modeling and CFD modeling. The bulk-flow modeling for honeycomb and hole-pattern seals is based on the Hirs model for turbulent lubricant films to obtain the rotor-dynamic coefficients, which has high computational efficiency [17]. Nelson [18] first provided analysis that predicts stiffness and damping coefficients. Kleynhans and Childs [19] review the various improvements to this theory. However, the accuracy of the bulk-flow modeling largely depends on empirical correlations that may vary with seal geometry and operating conditions [20]. Compared with the bulk-flow modeling, the CFD modeling solves the full Navier-Stokes equations, and provides a great deal of insights to understand the fluid dynamics of the annular gas seal flows than bulk flow modeling [21]. Some researchers have applied the CFD method to predict the leakage rates and rotor-dynamic coefficients of damper seals. Chochua et al. [4] and Nielsen et al. [22] employed a uni-axial whirl approach to acquire the rotor-dynamic coefficients of hole-pattern damper seals. Yan et al. [23,24] obtained the rotor-dynamic coefficients of hole-pattern damper seals by circular and elliptical whirl approaches from the uniaxial whirl approach. The above CFD methods used the single-frequency whirl model, which requires independent evaluation for each whirl frequency component of the rotor whirl motion. To improve computational efficiency with the acceptable predicting accuracy, Li et al. [25] proposed a multi-frequency elliptical whirl approach to obtain the rotor-dynamic coefficients of hole-pattern damper seals in a large frequency range. 

The above experimental measurements and numerical modeling discuss constant clearance (i.e, the same radial clearance for inlet and outlet) hole-pattern damper seals. However, in real engineering, the constant clearance damper seals generally turn into convergent-tapered (i.e., larger radial clearance for inlet relative to outlet) hole-pattern damper seals or divergent-tapered (i.e., less radial clearance for inlet with respect to outlet) hole-pattern damper seals due to a manufacturing error and elastic distortion caused by high temperature and high pressure gas. Vannini et al. [26] numerically and experimentally showed that convergence clearance damper seals increase effective stiffness coefficients, and generates a positive static stiffness when the rotor has eccentricity. Camatti et al. [27] and Tecza et al. [28] attributed a back-to-back compressor with rotor-dynamic instability on the test bench to seal a clearance divergence on damper seals. The high tapering sensitivity adopted in a straight-through compressor was instability because dirt gas clogs the damping seal and produces a divergent seal clearance [29].

To study the influence mechanism of taper seal clearance on the static and rotor-dynamic characteristics of hole-pattern damper seals, the three-dimensional transient computational fluid dynamic methods, including single-frequency whirl model and multi-frequency whirl model, are developed by a transient solution combined with the mesh deformation technique. Therein, the single-frequency whirl model is used to investigate the influence of taper degrees on the reaction forces and rotor-dynamic coefficients, while the multi-frequency whirl model is employed to calculate the frequency dependent rotor-dynamic coefficients of hole-pattern damper seals in a large frequency range. The streamlines, pressure distribution features, and the influence of taper degrees on the leakage rates are also discussed.

## 2. Theoretical Models for the Rotor-Dynamic Coefficients of Annular Gas Seals

### 2.1. The Linear Model of the Reaction Force and Rotordynamic Coefficients 

For a small relative motion of the rotor to stator centered within annular gas seals, the relationship between reaction forces $\left(F_{x},F_{y}\right)$ and frequency-dependent rotor-dynamic coefficients (K(Ω),k(Ω),C(Ω),c(Ω)) are [30,31]:

(1) $$-\left[\begin{array}{l} F_{x}\\ F_{y} \end{array}\right]=\left[\begin{array}{ll} K(\Omega ) & k(\Omega )\\ -k(\Omega ) & K(\Omega ) \end{array}\right]\left[\begin{array}{l} x\\ y \end{array}\right]+\left[\begin{array}{ll} C(\Omega ) & c(\Omega )\\ -c(\Omega ) & C(\Omega ) \end{array}\right]\left[\begin{array}{l} x^{\prime }\\ y^{\prime } \end{array}\right],$$

where (x,y) and $\left(x^{\prime },y^{\prime }\right)$ are whirl displacements and whirl velocities of the rotor, respectively. C(Ω) is the direct damping coefficient. c(Ω) is the cross-coupled damping coefficient. k(Ω) is the cross-coupled stiffness coefficient. K(Ω) is the direct stiffness coefficient.

As for the rotor-dynamic coefficients of annular gas seals, the direct damping coefficient C(Ω) is a resistance force of rotor whirl motion, i.e., larger C(Ω) induces higher energy consumption for the rotor whirl. The cross-coupled damping coefficient c(Ω) has a weak influence on the stability of a rotor system. k(Ω) is the source of rotor whirl motion. Increasing k(Ω) results in a more unstable rotor system when C(Ω) does not increase. The direct stiffness coefficient K(Ω) reflects the effect of gas on the natural frequency of the rotor system, and the natural frequency increases for positive K(Ω).

To comprehensively reflect the effect of annular gas seals on rotor system stability, the effective stiffness coefficient *K_eff_* and effective damping coefficient *C_eff_* are defined in Equation (2). Larger *K_eff_* and *C_eff_* are beneficial for the stability of the rotor system [19].

(2) $$\left\{ \begin{array}{l} K_{eff}=K+\Omega \cdot c\\ C_{eff}=C-\frac{k}{\Omega } \end{array}\right.,$$

### 2.2. Mathematical Expressions of Rotor Whirl Orbit

**Figure 1:**
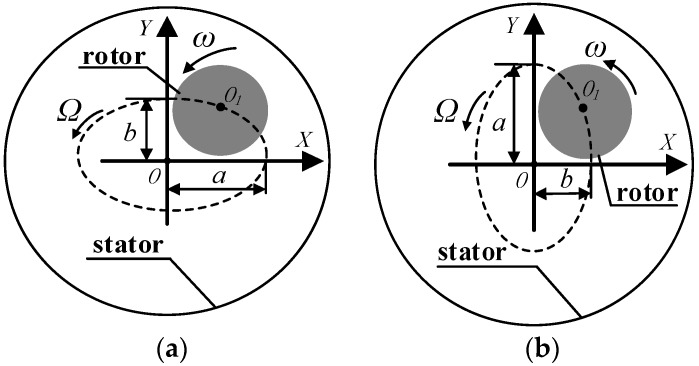
Rotor motion orbits in seal. (**a**) The long axis of an ellipse is on the *x*-axis. (**b**) The long axis of an ellipse is on the *y*-axis.

The rotor motion orbits in the seal is shown in Figure 1. Ideally, the rotor center is concentric with the seal stator center, and the rotor only rotates around the stator center with the rotational speed *ω*. However, in real turbomachinery, the rotor center orbits the stator center with an angular velocity Ω due to rotor quality eccentricity or other external disturbing forces. 

The equations for the rotor whirl are below.

(3) $$\left\{ \begin{array}{l} x=b\sin(\Omega t)\\ y=a\cos(\Omega t) \end{array}\right.,$$

(4) $$\left\{ \begin{array}{l} x=a\cos(\Omega t)\\ y=b\sin(\Omega t) \end{array}\right.,$$

where Ω=2πf, in which *f* is the rotor whirl frequency. *a* and *b* are whirl amplitudes of the rotor, and the major axis and minor axis of the elliptical orbit are on the coordinate axis. When a≠b>0, a=b>0 and b>a=0 or a>b=0, Equations (3) and (4) represent elliptical orbits, circular orbits, and sinusoidal line orbits, respectively. However, the actual whirl orbit of the rotor is closer to an elliptical orbit due to rotor misalignment and the difference of rotor-dynamic characteristics between vertical and horizontal directions for journal bearing.

### 2.3. Single-Frequency Elliptical Orbit Whirl Model

Single-Frequency Elliptical Orbit Whirl Model (SFWM) indicates the rotor center orbit and the seal stator center *O* with an elliptical orbit. The rotor-dynamic coefficients of annular gas seals are obtained when the rotor whirl is governed by Equation (4). The *a* = 0.1*C_r_* and *b* = 0.05*C_r_* are the whirl amplitudes of the rotor in the *x* and *y* direction, respectively, in which *C_r_* is the seal radial clearance. To simplify the calculation, the whirl displacements and whirl velocities are obtained if the timesteps *Ωt* = 0 deg and *Ωt* = 90 deg are selected in a transient computation. The obtained whirl displacements and whirl velocities are inputted into Equation (1). The relationship between reaction forces and rotor-dynamic coefficients of annular gas seals can be expressed as Equations (5) and (6), respectively. With respect to Equations (5) and (6), therefore, the frequency dependent rotor-dynamic coefficients of annular gas seals are obtained.

When Ωt=0°,

(5) $$\left\{ \begin{array}{l} K\cdot a+c\cdot b\cdot \Omega =-\Delta F_{x1}\\ -k\cdot a+C\cdot b\cdot \Omega =-\Delta F_{y1} \end{array}\right.,$$

When Ωt=90°,

(6) $$\left\{ \begin{array}{l} k\cdot b-C\cdot a\cdot \Omega =-\Delta F_{x}{}_{2}\\ K\cdot b+c\cdot a\cdot \Omega =-\Delta F_{y2} \end{array}\right.,$$

### 2.4. Multi-Frequency Elliptical Orbit Whirl Model

The Multi-Frequency Elliptical Orbit Whirl Model (SFWM) needs a separate transient computation to obtain the rotor-dynamic coefficients of annular gas seals for each whirl frequency, so that the computing time significantly increases, especially for the calculation of frequency dependent rotor-dynamic coefficients in a large frequency range. Thereby, MFWM is adopted to enhance the efficiency. In MFWM, the reaction forces at a specific frequency are caused by rotor whirl motion excitation with the same frequency. Linear superposition assumption for rotor whirl motion and the reaction force is soundness and is the basis theory for MFWM [20]. For MFWM, rotor whirl motion is defined as multiple harmonic functions with specific amplitudes. The whirl equation of the rotor with MFWM are defined below.

(7) $$\left\{ \begin{array}{l} x=c\cdot \sum_{i=1}^{N}\cos(\Omega_{i}t)\\ y=d\cdot \sum_{i=1}^{N}\sin(\Omega_{i}t) \end{array}\right.,$$

where *N* are the number of rotor whirl frequencies, $\Omega_{i}=2\pi f_{i}$ is the *i*th whirl angular velocity, *f_i_* is the *i*th whirl frequency, *c* = 0.01*C_r_* and *d* = 0.005*C_r_* are the whirl amplitudes for each frequency component in the *x* and *y* direction, respectively. Besides, it is difficult to distinguish the whirl displacements and reaction forces of each frequency component in the time domain because they are multi-frequency signals. In light of the Fast Fourier Transform (FFT) algorithm [32] and Equation (1), the linear model of annular gas seals with MFWM are shown below.

(8) $$-\left[\begin{array}{l} F_{x}(j\Omega )\\ F_{y}(j\Omega ) \end{array}\right]=\left[\begin{array}{ll} D(j\Omega ) & E(j\Omega )\\ -E(j\Omega ) & D(j\Omega ) \end{array}\right]\left[\begin{array}{l} X(j\Omega )\\ Y(j\Omega ) \end{array}\right],$$

where,$j=\sqrt{-1}$, D(jΩ)=K(Ω)+jΩ⋅C(Ω), and E(jΩ)=k(Ω)+jΩ⋅c(Ω) are impedance coefficients of annular gas seals. The impedance coefficients are complex numbers and contain multiple frequency components, i.e.,

(9) $$\left\{ \begin{array}{l} D(j\Omega )=\frac{-F_{x}(j\Omega )\cdot X(j\Omega )-F_{y}(j\Omega )\cdot Y(j\Omega )}{X^{2}+Y^{2}}\\ E(j\Omega )=\frac{-F_{x}(j\Omega )\cdot Y(j\Omega )+F_{y}(j\Omega )\cdot X(j\Omega )}{X^{2}+Y^{2}} \end{array}\right.,$$

Herein, the stiffness and damping coefficients of annular gas seals correspond with the real and imaginary parts of *D*(*jΩ*) and *E*(*jΩ*), respectively, i.e.,

(10) {K(Ω)=Re(D(jΩ))k(Ω)=Re(E(jΩ))C(Ω)=Im(D(jΩ))Ωc(Ω)=Im(E(jΩ))Ω,

From the above analysis, the frequency-dependent rotor-dynamic coefficients of annular gas seals in a large frequency range are obtained by an independent transient computation with MFWM.

## 3. Numerical Model for the Rotor-Dynamic Coefficients of Annular Gas Seals

### 3.1. Seal Geometry

**Figure 2:**
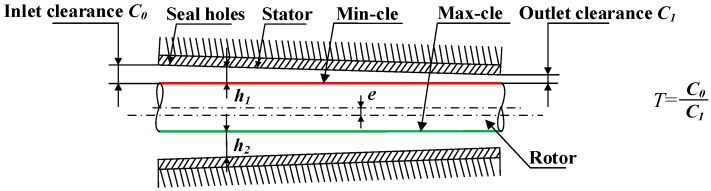
Schematic diagram of pressure extraction for minimum and maximum sealing clearance.

**Figure 3:**
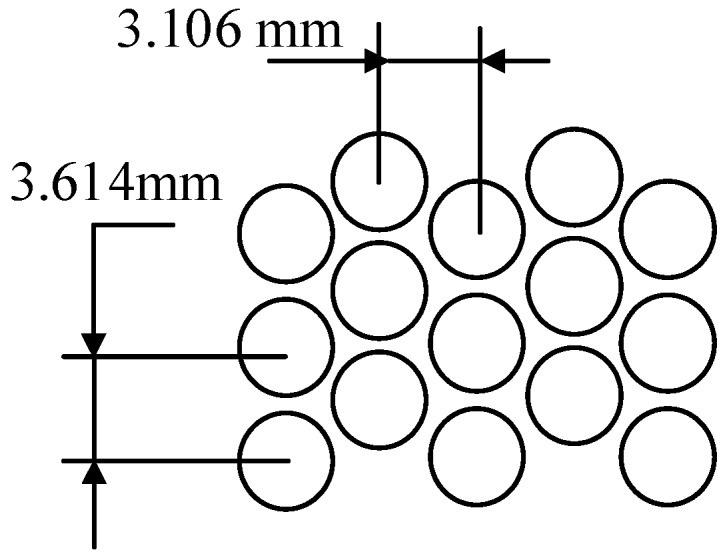
Distribution of seal holes in the stator surface.

To compare with taper clearance hole-pattern damper seals, the constant clearance damper seal in the work of Childs et al. [15] is adopted as one object of study. As shown in Figure 2, the taper degree *T* is defined as the ratio of inlet radial clearance to exit radial clearance, in which *T* = 1, *T >* 1, and *T* < 1 indicate constant clearance damper seals, convergent-tapered damper seals, and a divergent-tapered damper seal, respectively. The maximum clearance (max-cle) and minimum clearance (min-cle) after rotor eccentricity are also illustrated in Figure 2. The geometry parameters and operating conditions of constant clearance damper seals in Reference [15] are shown in Table 1. The seal holes in the stator surface are arranged in a staggered array, as shown in Figure 3. Nine taper degrees (3, 2.5, 2, 1.5, 1, 0.66, 0.5, 0.4, 0.33) are discussed. The 0.2 mm is the minimum radial clearance of taper clearance hole-pattern damper seals. Geometry parameters and operating conditions for eight taper clearance hole-pattern damper seals besides inlet or outlet radial clearance, are consistent with constant clearance damper seals in Reference [15].

### 3.2. Computational Grid

**Figure 4:**
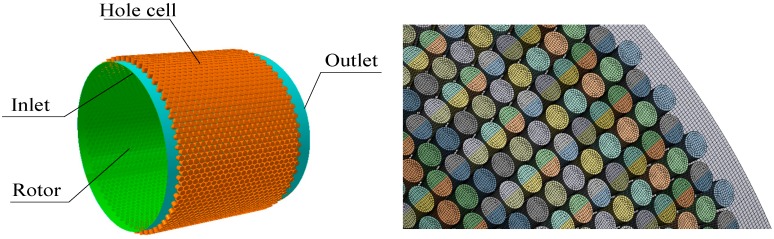
Computational model and meshing of constant clearance damper seals.

The whole circumferential modeling and meshing are required since the rotor whirl is an off-centered process. The computational model and the part with high quality hexahedral grids of the constant clearance hole-pattern damper seal are shown in Figure 4. Seal clearance is an important design parameter for determining operating efficiency and rotor stability. The seal clearance is very small relative to the diameter and width of the hole-pattern damper seal. To capture high speed jet flow at seal clearance, the grid density of seal clearance should be larger. Table 2 explains the relationship between leakage rates and the number of grid elements. Through this grid analysis, we find that the relative error of leakage rates caused by the number of elements is less than 1% when the number of elements is greater than 6.9 × 10^6^, and the minimum orthogonality of the grid is greater than 51.9 deg and the maximum aspect ratio is 44 as well. The results explain that the quality of the grid satisfies the requirement of seal design and characteristic analysis. Therefore, the final number (6.9 × 10^6^) of elements is adopted in this paper.

### 3.3. Numerical Method

In this work, the rotor whirl frequency range (40–320 Hz) is investigated. Due to the differences between rotational timescale and the whirl timescale as well as the need to correctly capture rotational and whirl features, the timesteps used for the SFWM are considered to be variable with rotor whirl frequency. It is reasonable to select the timesteps ranging from 30 to 100 per period of the rotor whirl25. To guarantee the accuracy of analysis results, 100 timesteps are considered during one period for each whirl frequency in this study.

For MFWM, the whirl displacements and reaction forces are multi-frequency signals. The timestep used for transient analysis is determined by the highest rotor whirl frequency, and, thereby, there are least 30 sampling points per period for the highest frequency. To guarantee the accuracy of whirl displacements and reaction forces, we assumed the sampling frequency of 10,000 Hz (timestep 0.0001 s) and transient computing time of 0.5 s. According to Equations (8)–(10), the rotor-dynamic coefficients for each rotor whirl frequency can be identified as long as the ratio of the sampling frequency to the number of data points is less than the difference of adjacent frequencies of the rotor whirl. In this work, the first 2000 data points in iterative curves of rotor whirl displacements and reaction forces are selected. The frequency resolution of whirl displacements and reaction forces is 5 Hz, which is far less than the difference (40 Hz) of adjacent rotor whirl frequencies. Therefore, the rotor-dynamic coefficients for each rotor whirl frequency can be identified.

The initial flow field is gained in transient analysis by a steady analysis of the motion model at a rotational speed. The desired convergence target of steady solution is that the root mean square residuals of the momentum and mass equations, energy equations, and turbulence equations reach a value of 10^−6^, and the overall imbalance of the mass is less than 0.1%. For the transient analysis, the reaction forces on the rotor surface approach to periodic consecutive cycles after a half-period, and the desired convergence target of transient analysis is that the difference of reaction forces between the adjacent two cycles should be less than 0.2% when the rotor whirls to the same position in the sealing cavity.

### 3.4. Boundary Conditions

3D Reynolds-Averaged Navier-Stokes (RANS) equations with standard *k-ε* turbulence model are used to gain the rotor-dynamic coefficients of hole-pattern damper seals in the present paper. The rotor position is physically perturbed in a periodic whirl orbit using moving mesh techniques, and a mesh displacement diffusion model is adopted to govern mesh deformation. The air ideal gas at 17.4 °C and 1 atmospheric pressure is used with density of 1.2147 kg/m^3^ and dynamic viscosity of 1.1831 × 10^−5^ kg/(m·s). Since the temperature changed only 3.3 K through the constant clearance hole-pattern damper seal [15], the isothermal model is adopted. The total pressure, total temperature, and turbulence quantities are defined (turbulent intensity = 5%) at the inlet, while static pressure is appointed at the outlet. The wall surfaces are defined to be smooth and adiabatic, and the near wall velocity is described by the scalable logarithmic wall function. The high-resolution scheme and the second order backward Euler scheme were applied for the spatial discretization and the transient discretization, respectively. 

The numerical calculations were conducted by using the commercial software ANSYS CFX16.0. On the hardware, one Intel Xeon E5-2650 v3 2.3 GHz CPU (Intel Corporation, Santa Clara, CA, USA) computer with 64 GB RAM was used. The computation time was about 72 hours and 168 hours for a transient analysis of SFWM and MFWM, respectively.

## 4. Results and Discussion

### 4.1. Streamlines and Pressure Distribution Features of Hole-Pattern Damper Seals

**Figure 5:**
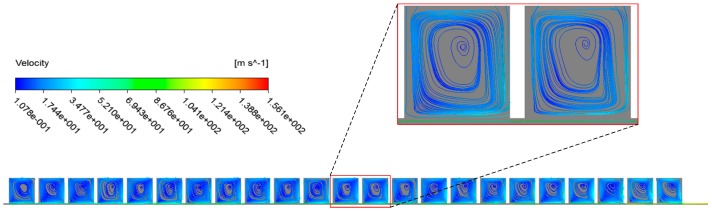
Two-dimensional streamlines on the midplane of the seal.

2D streamlines on the midplane of constant clearance damper seals, which is shown in Figure 5. As revealed in Figure 5, one part of gas directly goes through seal clearance and the other part forms a big vortex as a result of rotor high speed rotation and pressure difference between the inlet and the outlet. Numerous seal holes in the stator surface lead to a large reduction of gas forward speed, which is promising for reducing leakage rates of hole-pattern damper seals. The gas in seal cavities anti-rushes to the seal clearance and chokes the oncoming gas to further decrease leakage rates.

**Figure 6:**
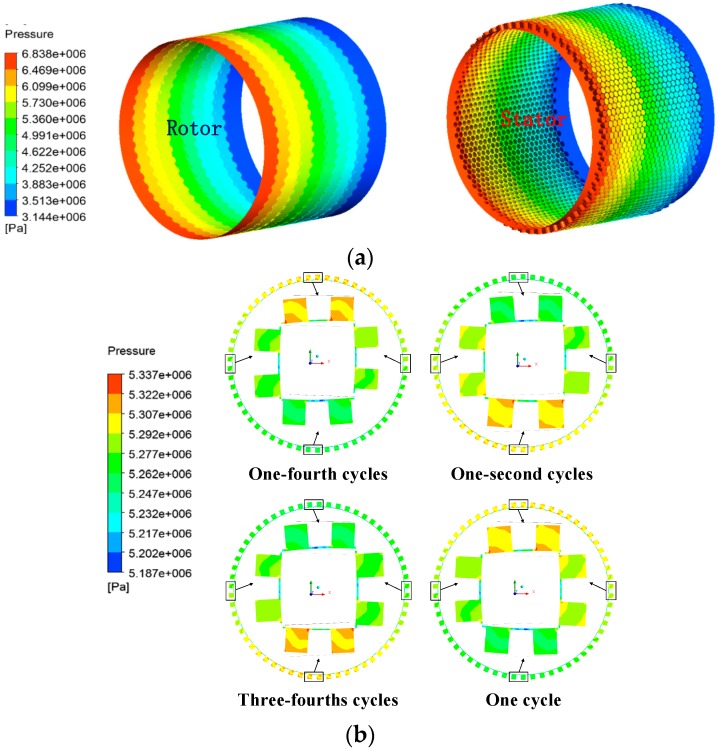
Pressure distribution features. (**a**) Steady pressure distribution features of rotor and stator surfaces (*T* = 1.5 and min-cle = 0.2 mm). (**b**) Transient pressure distribution features (*T* = 1.5 and min-cle = 0.2 mm).

The pressure distribution features of constant clearance damper seals in steady analysis are drawn in Figure 6a. It indicates that the static pressure is almost constant below the hole chamber, but quickly decreases under the tooth due to the leakage jet. Because the leakage flow under the tooth transfers its internal energy to kinetic energy in the seal clearance, the velocity of gas flow quickly increases and causes a drop in pressure. After gas flow passed seal clearance, the sudden increase of the flow area causes a large vortex in the hole chamber region where the velocity of gas flow decreases and the kinetic energy is transferred into the internal energy. Figure 6b shows transient pressure distribution features on a schematic cross section of the *XY* plane for the convergent-tapered (*T* = 1.5) damper seal in a period of rotor whirl motion. It is shown that the static pressure is unbalanced along the circumferential direction on the rotor surface due to different whirl positions of the rotor, and periodical change of seal clearance causes the variation of unbalanced pressure with time.

### 4.2. Influence of Taper Degrees on the Leakage Rate of the Hole-Pattern Damper Seal

**Figure 7:**
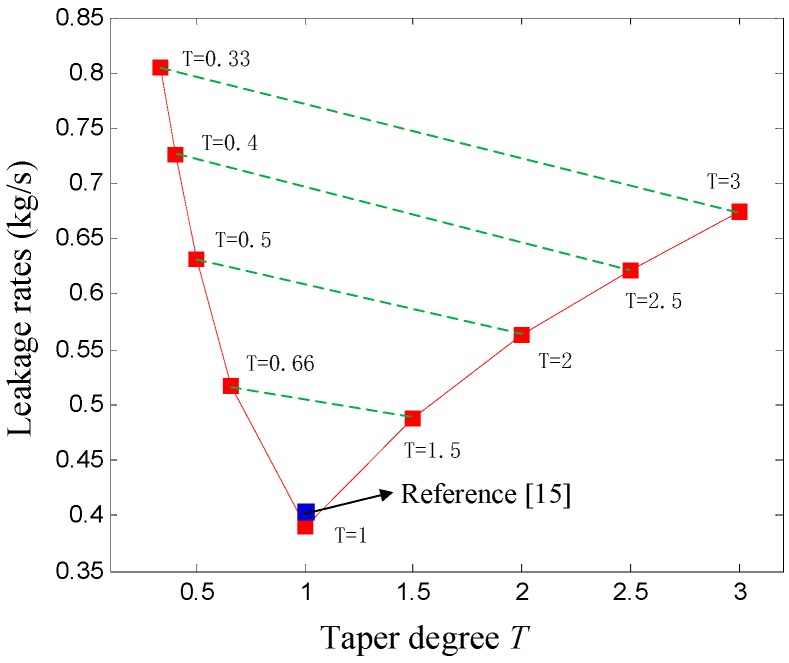
Influence of taper degrees *T* on the leakage rate.

**Figure 8:**
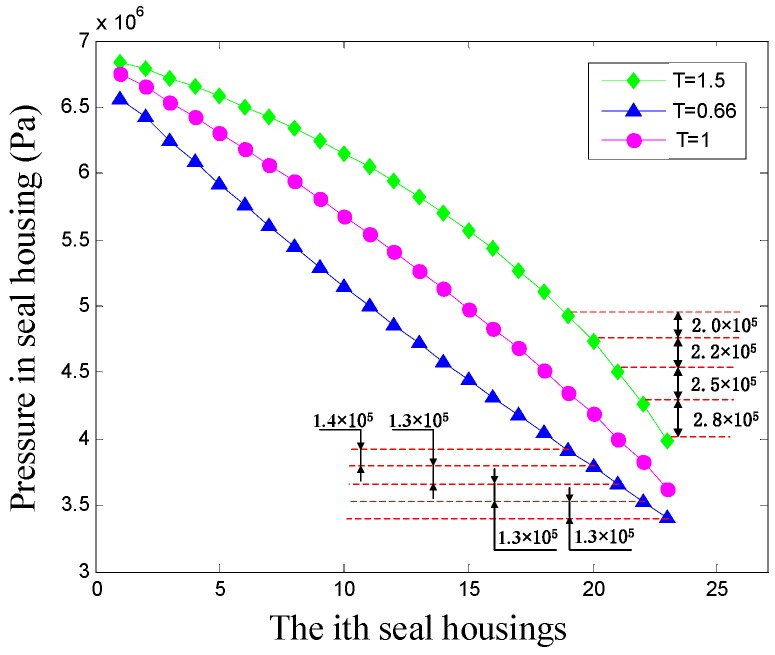
Pressure distribution features of taper clearance damper seals.

Figure 7 shows the relationship between taper degrees and leakage rates of hole-pattern damper seals. The dotted line connection represents the same average seal clearance (the half sum of inlet and outlet radial clearance) for divergent-tapered (*T* = 0.33, 0.4, 0.5, 0.66) and divergent-tapered (*T* = 3, 2.5, 2, 1.5) hole-pattern damper seals. It is shown that the leakage rates of hole-pattern damper seals increase with the rise of average seal clearance. Relative to divergent-tapered damper seals, the leakage rates of convergent-tapered damper seals are less for the same average seal clearance. The leakage rates of four convergent taper degrees (*T* = 3, 2.5, 2, 1.5) are 16.16%, 14.35%, 10.77%, and 5.39% less than the other four divergent taper degrees (*T* = 0.33, 0.4, 0.5, and 0.66). Figure 8 shows the pressure curves in each seal cavity of the hole-pattern damper seal for *T* = 0.66, *T* = 1, and *T* = 1.5. The results in Figure 8 illustrate that the pressure at the same seal housing positively increases with the growth of taper degrees. Larger pressure drops located on the adjacent side of the seal outlet causes less leakage rates for the hole-pattern damper seals. The pressure drop at *T* = 1.5 is larger than *T* = 0.66 at the same seal housing near the seal outlet, and there is no pressure change in the last four seal cavities for *T* = 0.66. Therefore, the leakage rates of convergent-tapered damper seals are less than these of divergent-tapered damper seals for the same average seal clearance.

### 4.3. Whirl Displacement and Reaction Force of Hole-Pattern Damper Seal

**Figure 9:**
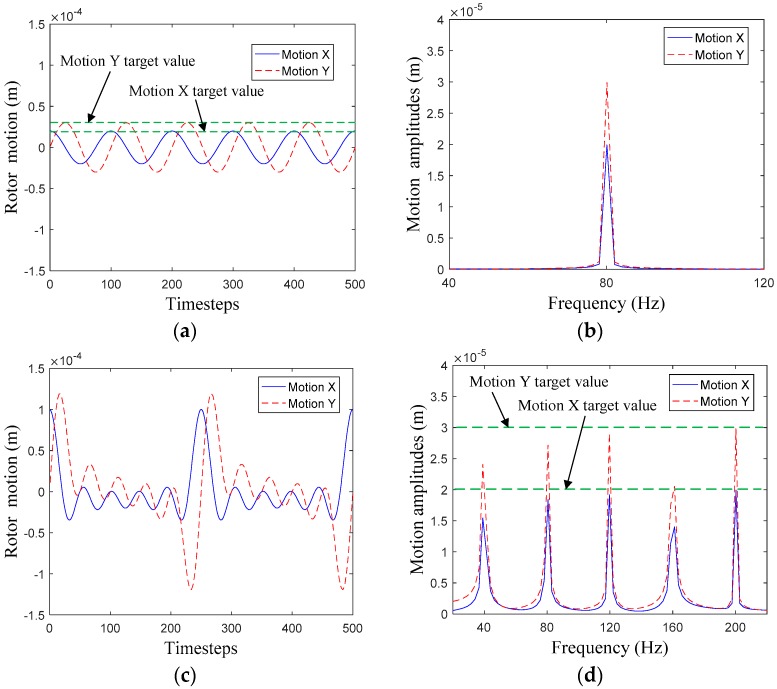
Whirl displacements of the rotor with SFWM and MFWM. (**a**) Time domain with SFWM. (**b**) Frequency domain with SFWM. (**c**) Time domain with MFWM. (**d**) Frequency domain with MFWM.

**Figure 10:**
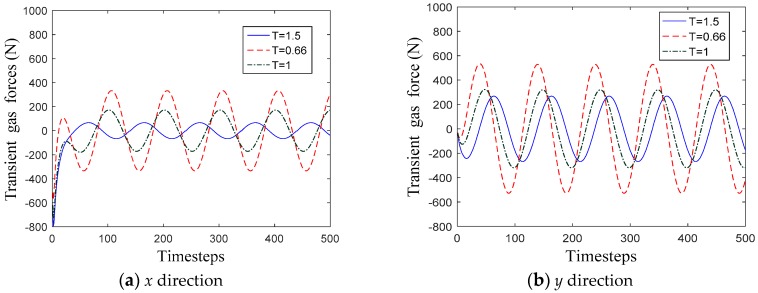
Transient gas forces for three taper degrees using SFWM. (**a**) *x* direction; (**b**) *y* direction.

Rotor whirl displacements with SFWM and MFWM are illustrated in Figure 9. Reaction forces gained from SFWM are drawn in Figure 10 for *T* = 0.66, *T* = 1, and *T* = 1.5, respectively.

As shown in Figure 9, for SFWM and MFWM, the target values of rotor whirl frequencies and whirl amplitudes can be captured by the frequency spectrum, in which the error between two whirl models are caused by dynamic mesh and the FFT algorithm. However, the rotor whirl displacements have little impact on the rotor-dynamic coefficients when whirl amplitudes of the rotor meet the small perturbation condition. Compared to the MFWM, the SFWM more accurately capture rotor whirl amplitudes, so the SFWM is more applicable to investigate the relationship between rotor whirl and reaction forces, and the gas exciting-vibration mechanism as well.

As shown in Figure 10, compared with the constant clearance hole-pattern damper seal, peak values of reaction forces of the convergent-tapered hole-pattern damper seals are smaller at 61.78% and 16.23% in *x* direction and *y* direction, while peak values of divergent-tapered hole-pattern damper seals are larger at 89.89% and 67.22% in *x* and *y* directions. Thus, the taper degrees change reaction forces by acting on the rotor and further influence the stability of the rotor system.

### 4.4. The Relation between Whirl Frequency and Rotor-Dynamic Coefficients

**Figure 11:**
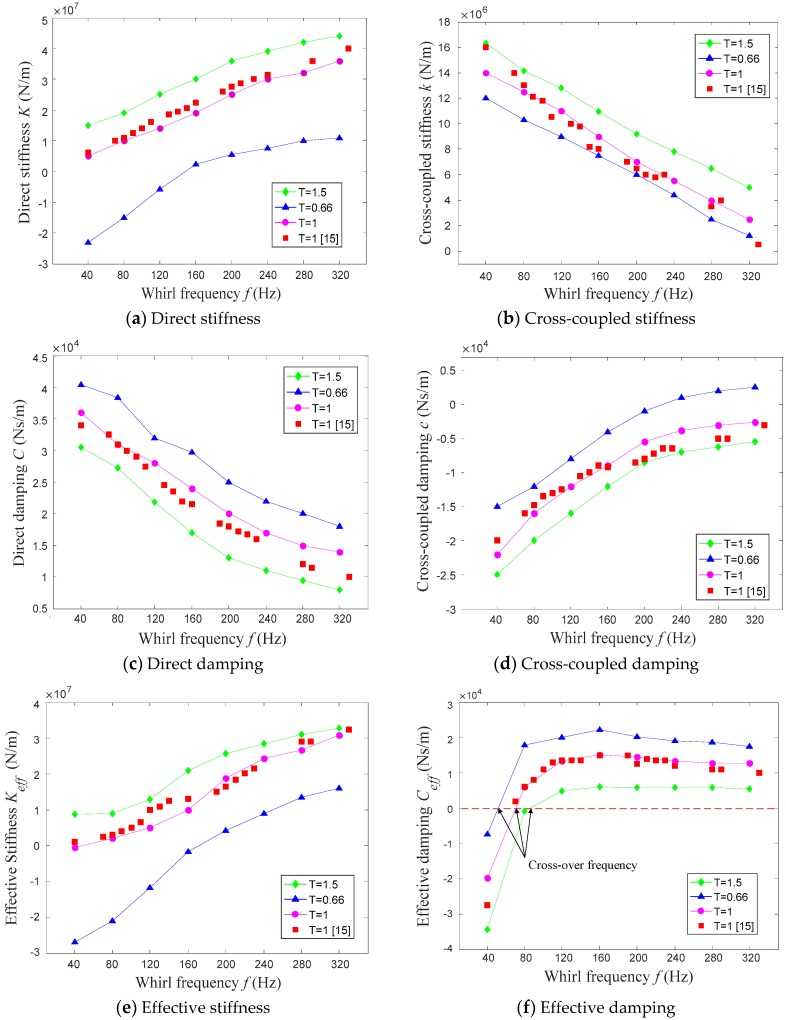
Effect of dynamic characteristics (**a**–**e**) of hole-pattern damper seals on whirl frequency, where N (*P_inlet_* = 7 MPa, *P_outlet_ =* 3.15 MPa, RS = 20,200 r/min, *T_inlet_ =* 17.4 °C).

Figure 11 shows the relationship between whirl frequency and the rotor-dynamic coefficient of the hole-pattern damper seals for *T* = 0.66, *T* = 1, and *T* = 1.5, respectively. 

As revealed in Figure 11, the taper degree does not influence the trend of rotor-dynamic coefficients versus whirl frequencies. The direct stiffness coefficient, the cross-coupled damping coefficient, the effective stiffness coefficient, and the effective damping coefficient rise with the increase of whirl frequency, while the opposite trend happens for the cross-coupled stiffness coefficient and the direct damping coefficient with the increase of whirl frequency. 

Cross-over frequency is the frequency that the effective damping coefficient transitions from a negative value to a positive value with the increasing whirl frequency. In light of rotor-dynamic theory, annular gas seals provide negative damping when the cross-over frequency of annular gas seals is greater than the natural frequency of the rotor system, and it is harmful to the rotor system when the rotor passes the critical speed. From Figure 11f, comparing with the constant clearance hole-pattern damper seal, the cross-over frequency of the convergent-tapered hole-pattern damper seals increase by 22.43%, while the cross-over frequency of divergent-tapered hole-pattern damper seals decreases by 26.91%.

### 4.5. Influence of Taper Degree on Natural Frequency of the Rotor System

**Figure 12:**
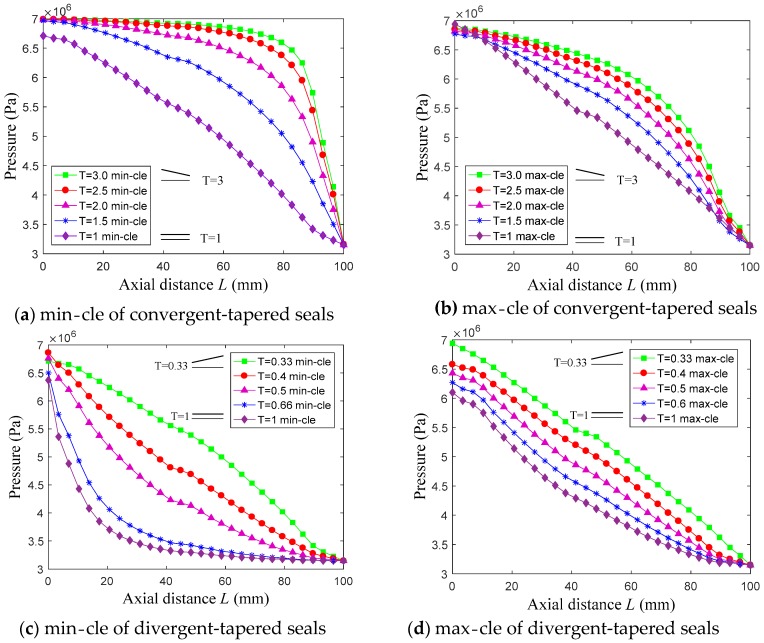
Pressure distribution of the rotor surface. (**a**) min-cle of convergent-tapered seals; **(b)** max-cle of convergent-tapered seals; (**c**) min-cle of divergent-tapered seals; (**d**) max-cle of divergent-tapered seals.

Figure 12 shows the pressure distribution curves of minimum clearance (min-cle) and maximum clearance(max-cle) on the rotor surface along the axial direction for nine taper degrees when the rotor eccentricity ratio is 0.8.

As illustrated in Figure 12, the pressure on the rotor surface of convergent-tapered damper seals are larger than constant clearance damper seals at the same axial location, while the pressure of divergent-tapered damper seals are less than constant clearance damper seals. Constant clearance damper seals have a small pressure difference between min clearance and max clearance due to the Lomakin effect [33], while taper clearance hole-pattern damper seals are large. For convergent-tapered damper seals, the axial pressure drops gradually and increases close to the seal outlet as the ratio between the inlet and outlet clearance increases. This is due to a small clearance near the seal outlet, which results in higher velocity, and, hence, higher pressure drops. Additionally, divergent-tapered damper seals have the opposite trend. Hence, convergent-tapered damper seals have a large positive direct stiffness coefficient because the pressure at small seal clearance is greater than that at large seal clearance, which improves the natural frequency of the rotor system and provides large static load capacity by a rotor running eccentric. However, divergent-tapered damper seals produce large negative direct stiffness, which decrease the natural frequency of the rotor system, and it is not beneficial for rotor stability because the natural frequency of the rotor system is less than the cross-over frequency of annular gas seals.

In line with the above analysis, when rotor has eccentricity, convergent-tapered damper seals significantly increase the natural frequency of the rotor system and provide static load rather than a constant clearance damper seal and divergent-tapered damper seals. Therefore, convergent-tapered damper seals hold the functions of both sealing and journal bearing, which is helpful for the stability of the rotor system when rotor eccentricity is the main fact of rotor instability.

### 4.6. Influence of Taper Degrees on Rotordynamic Coefficients of the Hole-Pattern Damper Seal

**Figure 13:**
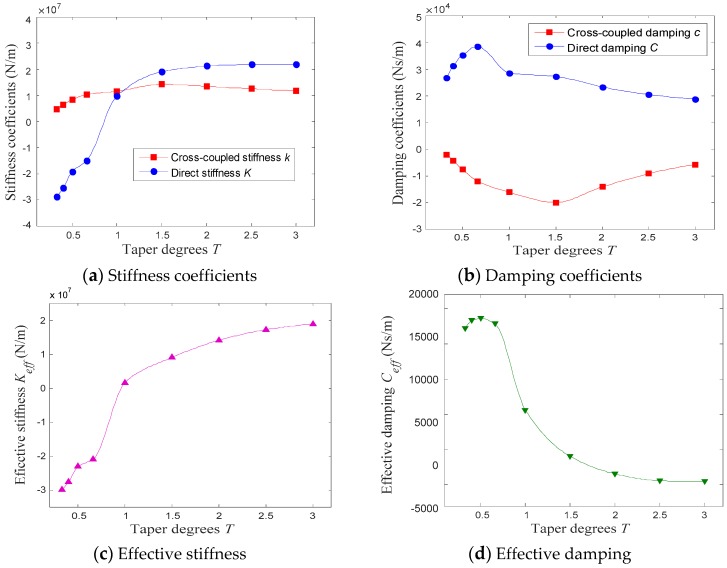
The influence of taper degrees on rotor-dynamic coefficients (**a**–**d**) of hole-pattern damper seals, where *P_inlet_* = 7 MPa, *P_outlet_* = 3.15 MPa, RS = 20,200 r/min, and *T_inlet_* = 17.4 °C.

Figure 13 shows the relationship between taper degrees and rotor-dynamic coefficients when rotor whirl frequency is 80 Hz.

As shown in Figure 13, taper degrees largely influence the rotor-dynamic coefficients of hole-pattern damper seals. Compared with the constant clearance hole-pattern damper seal, the maximum relative variation of the direct stiffness coefficient, cross-coupled stiffness coefficient, direct damping coefficient, cross-coupled damping coefficient, effective stiffness coefficient, and effective damping coefficient are 391.37%, 59.42%, 34.94%, 87.38%, 1865.38%, and 236.48%. Furthermore, with the increase of taper degrees, the cross-coupled stiffness coefficient*,* the direct damping coefficient, and the effective damping coefficient are first increased, then decreased, and, lastly, reached the maximum near *T* = 1.5, *T* = 0.66, and *T* = 0.5, respectively. The direct stiffness coefficient first increases, then slightly decreases, and, lastly, reach the maximum near *T* = 2.5. The cross-coupled damping coefficient first decreases and then increases, and, lastly, reach the minimum near *T* = 1.5. An effective stiffness coefficient increases with the growing taper degrees.

## 5. Conclusions

When the transient solution is combined with a mesh deformation technique, both the single-frequency whirl model and the multi-frequency whirl model are established for the static and rotor-dynamic characteristic analyses of convergent-tapered and divergent-tapered hole-pattern damper seals. In term of the investigations, some conclusions are summarized below.

(1) Taper seal clearance increases leakage rates when the minimum seal clearance is the same for taper clearance hole-pattern damper seals and the constant clearance hole-pattern damper seal. The leakage rates of convergent-tapered damper seals are less than divergent-tapered damper seals for the same average seal clearance due to a larger pressure drop at the same seal housing near the seal outlet. The maximum relative variation of leakage rates reaches 16.16% in this paper.

(2) The taper degrees change the reaction forces and rotor-dynamic coefficients of hole-pattern damper seals. Compared with the constant clearance hole-pattern damper seal, the maximum relative variation of the rotor-dynamic coefficients is 1865.38% for nine taper degrees in this paper. The convergent-tapered damper seals have smaller reaction forces and effective damping coefficient *C_eff_*, larger cross-over frequency, and direct stiffness coefficient *K*, while the divergent-tapered damper seals have the opposite effects.

(3) The constant clearance damper seals have a small pressure difference between the minimum clearance and the maximum clearance due to the Lomakin effect, while the taper clearance hole-pattern damper seals are large. For the convergent-tapered damper seals, the axial pressure drops, which closes to the seal outlet as the ratio between the inlet and the outlet clearance gradually increases. The reason is that a small clearance near the seal outlet results in higher velocity and a higher pressure drop. The divergent-tapered damper seals have the opposite trend.

(4) The divergent-tapered damper seals alleviate rotor whirl motion due to a larger effective damping coefficient when the rotor system has large natural frequency (the natural frequency of the rotor system is greater than the cross-over frequency of divergent-tapered damper seals) and small eccentricity. The convergent-tapered damper seals increase the natural frequency of the rotor system and provide static load, which is promising for improving the stability of the rotor system when rotor eccentricity is the main factor causing rotor instability.

## Figures and Tables

**Table 1:** Basic parameters of constant clearance damper seals in Reference [15].

Parameters	Values	Parameters	Values
Seal length (mm)	85.7	Inlet pressure (MPa)	7.0
Rotor diameter (mm)	114.7	Inlet temperature (°C)	17.4
Seal clearance (mm)	0.2	Inlet preswirl	0
Hole diameter (mm)	3.302	Outlet pressure (MPa)	3.15
Hole numbers	2668	Rotor speed (r/min)	20,200
Hole area ratio (%)	69	Whirl frequencies (Hz)	40,80,120,160,200

**Table 2:** Influence of the number of grid elements on leakage rates.

Number of Elements (×10^6^)	Leakage Rates (kg/s)	Relative Error Compared with Experiment [15] (%)
3.2	0.345	15.65
4.7	0.370	9.54
5.5	0.380	7.09
6.9	0.390	4.65
8	0.392	4.16
9.76	0.393	3.91

## Nomenclature

*P =* complex variables

A = constant complex number

*a_1_* = constant real number

*a_2_* = constant real number

*b_1_* = constant real number

*b_2_* = constant real number

*a* = the major axis of journal single frequency elliptical orbit whirl (m)

*b* = the minor axis of journal single frequency elliptic orbit whirl (m)

*c* = the major axis of journal multiple frequencies elliptic orbit whirl (m)

*d* = the minor axis of journal multiple frequencies elliptic orbit whirl (m)

*K* = direct stiffness (N/m)

*k* = cross-coupling (N/m)

*C* = direct damping (N s/m)

*c* = cross-coupling damping (N s/m)

*K_eff_* = effective stiffness (N/m)

*C_eff_* = effective damping (N s/m)

*F_x_* = rotor response force in the *x* direction (N)

*F_y_* = rotor response force in the *y* direction (N)

Δ*F_x_*_1_ = the variation values of the rotor response force in the *x* direction at a certain timestep (N)

Δ*F_x_*_2_ = the variation values of the rotor response force in the *x* direction at a certain timestep (N)

Δ*F_y_*_1_ = the variation values of the rotor response force in the *y* direction at a certain timestep (N)

Δ*F_y_*_2_ = the variation values of the rotor response force in the *y* direction at a certain timestep (N)

*D* = impedance coefficients

*E* = impedance coefficients

*T* = taper degrees

*x*/*y* = rotor displacement in the *x*/*y* direction (m)

*x*′/*y*′ = rotor velocity in the *x*/*y* direction (m/s)

*f* = rotor whirl frequency (Hz)

*D* = stator diameter (mm)

*B* = journal width (mm)

*e =* the eccentricity distance of the rotor (m)

*C_r_* = sealing clearance (m)

*N* = the number of whirl frequencies

*Ω* = angular frequency of the rotor whirl (r/min)

*ω* = angular frequency of the rotor spin (rad/s)

*e* = eccentricity distance (mm)

*P_in_* = inlet pressure (Pa)

*P_out_* = outlet pressure (Pa)

RS = rotor speed (r/min)

*C_0_* = inlet clearance (mm)

*C_1_* = outlet clearance (mm)

*T_inlet_* = inlet temperature (°C)
